# Advancements in Cardiac CT Imaging: The Era of Artificial Intelligence

**DOI:** 10.1111/echo.70042

**Published:** 2024-11-25

**Authors:** Pietro Costantini, Léon Groenhoff, Eleonora Ostillio, Francesca Coraducci, Francesco Secchi, Alessandro Carriero, Anna Colarieti, Alessandro Stecco

**Affiliations:** ^1^ Department of Translational Medicine University of Eastern Piedmont Novara Italy; ^2^ Department of Biomedical Sciences and Public Health Marche Polytechnic University Ancona Italy; ^3^ Department of Biomedical Sciences for Health Università degli Studi di Milano Milano Italy; ^4^ Department of Radiology Unit of Cardiovascular Imaging IRCCS MultiMedica Sesto San Giovanni Italy

**Keywords:** artificial intelligence, computed tomography angiography, deep learning, epicardial adipose tissue, machine learning, myocardial fractional flow reserve, myocardial ischemia, myocardial perfusion imaging

## Abstract

In the last decade, artificial intelligence (AI) has influenced the field of cardiac computed tomography (CT), with its scope further enhanced by advanced methodologies such as machine learning (ML) and deep learning (DL). The AI‐driven techniques leverage large datasets to develop and train algorithms capable of making precise evaluations and predictions. The realm of cardiac CT is expanding day by day and multiple tools are offered to answer different questions. Coronary artery calcium score (CACS) and CT angiography (CTA) provide high‐resolution images that facilitate the detailed anatomical evaluation of coronary plaque burden. New tools such as myocardial CT perfusion (CTP) and fractional flow reserve (FFR_CT_) have been developed to add a functional evaluation of the stenosis. Moreover, epicardial adipose tissue (EAT) is gaining interest as its role in coronary artery plaque development has been deepened. Seen the great added value of these tools, the demand for new exams has increased such as the burden on imagers. Due to its ability to fast compute multiple data, AI can be helpful in both the acquisition and post‐processing phases. AI can possibly reduce radiation dose, increase image quality, and shorten image analysis time. Moreover, different types of data can be used for risk assessment and patient risk stratification. Recently, the focus of the scientific community on AI has led to numerous studies, especially on CACS and CTA. This narrative review concentrates on AI's role in the post‐processing of CACS, CTA, FFR_CT_, CTP, and EAT, discussing both current capabilities and future directions in the field of cardiac imaging.

## Graphical abstract

Artificial intelligence (AI) has nowadays entered the realm of cardiac computed tomography (CT) offering new tools. In this review, we focus on the application of AI in the post‐processing workflow for coronary artery calcium score, computed tomography angiography, fractional flow reserve CT, myocardial CT perfusion, and epicardial adipose tissue.

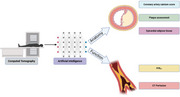

**Abbreviations**
AF: atrial fibrillation
AI: artificial intelligence
AUC: area under curve
CAC: coronary artery calcium
CACS: coronary artery calcium score
CAD: coronary artery disease
CAD‐RADS: Coronary Artery Disease‐Reporting and Data System
CFD: computational fluid dynamics
CHD: coronary heart disease
CNN: convolutional neural network
CT: computed tomography
CTA: computed tomography angiography
CTP: computed tomography perfusion
DL: deep learning
EAT: epicardial adipose tissue
FFRCT: fractional flow reserve computed tomography
ICA: invasive coronary angiography
LIE: late iodine enhancement
MACE: major adverse cardiovascular events
MBF: myocardial blood flow
ML: machine learning
ROC: receiver operating curve

## 1 Introduction

In the last decades, the request for cardiac computed tomography (CT) exams has steadily increased [1]. Cardiovascular diseases remain the leading cause of mortality globally [2], and non‐invasive imaging, such as cardiac CT, plays a crucial role in coronary artery disease (CAD) assessment.

The 2024 European Society of Cardiology guidelines emphasize the importance of computed tomography angiography (CTA) in both symptomatic and asymptomatic patients considering its high negative predictive value for excluding CAD [3]. CTA allows for valuable plaque assessment in which the grade of stenosis and the composition of plaque are easily depicted.

Lately, new tools have been developed to overcome the anatomical assessment and enter the functional one. Fractional flow reserve (FFR) is a known invasive technique to evaluate the impact a known coronary stenosis has on the coronary flow [4]. Recently, new technologies led to the possibility of a CT‐based evaluation of FFR, the so called FFR_CT_ [5]. This tool allows for non‐invasive FFR assessment and it has already proved to have an accurate prognostic value and to allow for better risk stratification [6]. Moreover, another promising tool that is rapidly gaining interest worldwide is CT perfusion (CTP). This technique revolves around assessing the myocardial enhancement in rest and stress (during pharmacological stress) to search for possible ischemic areas in which contrast enhancement is reduced. CTP can be acquired both in a static and dynamic setting. Static CTP is based on the acquisition of a single image during the administration of an iodinated contrast agent and allows for quality assessment. Dynamic CTP revolves on multiple acquisitions during contrasts infusion. The images are then processed to generate quantitative maps. Dynamic CTP proved to yield high diagnostic value for the incidence of major cardiovascular events [7]. Finally, a vivid interest is blooming in epicardial adipose tissue (EAT) evaluation, being the portion of fat between myocardium and the pericardium inner layer. Some studies highlighted how EAT yields a prognostic value towards major adverse cardiovascular events (MACE) [8].

Along with the development of cardiac CT, artificial intelligence (AI) has bloomed into the world of cardiac imaging.

AI refers to the capability of computational systems or algorithms to execute tasks traditionally requiring human cognitive functions. These tasks include reasoning, learning, problem‐solving, perception, and understanding natural language.

This goal is achieved through adaptation and analysis of big data sets. Usually, a developed AI algorithm can analyze and interpret multiple data in a shorter time compared to humans. Machine learning (ML) and deep learning (DL) are subsets of AI that focuses on automatic learning and improving (Table 1). The algorithm itself is programmed to find patterns, make decisions or predictions, and is based on neural networks. A neural network is composed of layers of interconnected nodes, called neurons or units, which process and transform data. ML focused on developing algorithms that allow computers to learn patterns and insights from data without being explicitly programmed for specific tasks. ML can shape itself when exposed to more data [9]. DL is a subfield of ML in which multiple layers of neural networks are developed to amplify its ability to automatically learn from large and complex datasets. Moreover, the neural networks used in DL are different. A convolutional neural network (CNN) is a deep neural network specifically designed for analyzing images, thus being very useful in the radiological world. Another one is backpropagation neural network (BPNN), a feedforward neural network that optimizes its weights and biases during training via error backpropagation [10].

**TABLE 1:** Brief description of the three major computational systems reviewed in the paper.

Term	Definition
AI	Umbrella definition that includes every computational algorithm that can mimic human cognitive functions, such as reasoning, learning, problem‐solving, perception [10].
ML	Subset of AI based on neural networks and capable of learn and adapt without following precise instructions [9].
DL	Subfield of ML, based on multiple neural networks, and capable of learning and adapting automatically from large datasets. DL models can learn intricate patterns and hierarchies of features. It uses different methods, such as CNN and BPNN [9, 11].

AI technologies are enhancing the interpretation of CT scans by improving image quality, reducing noise, automatically identifying key structures like coronary plaques, and aiding in the risk stratification of patients [12]. This review aims to delve into the many applications AI has in the post‐processing setting while investigating on the possible applications that will come.

## 2 Coronary Artery Calcium

**FIGURE 1:**
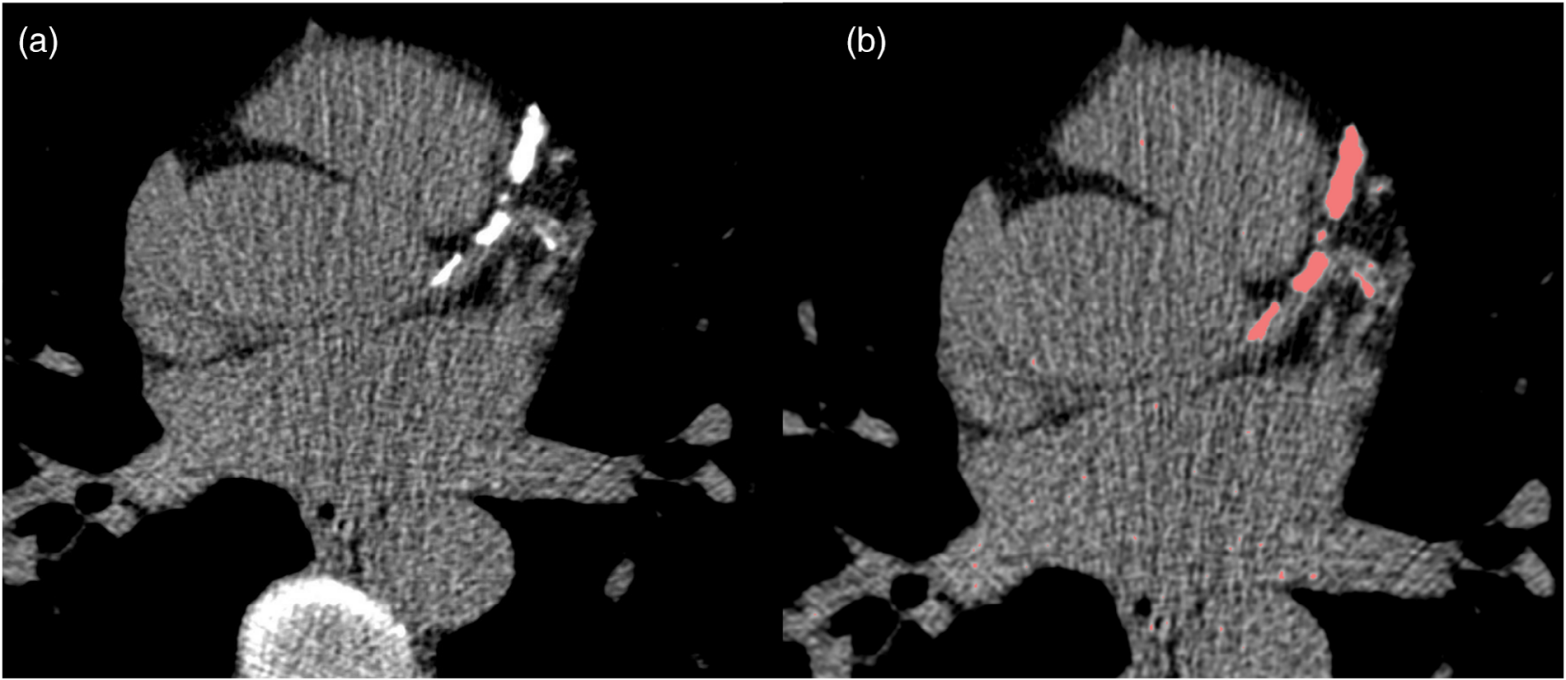
Example of an automatic measurement of CACS. Here an unenhanced CT scan is reported (a) along with the automatic segmentation of the coronary artery calcifications (b).

Coronary artery calcium score (CACS) is a quantitative evaluation of coronary artery calcium (CAC). The score is mediated through the Agatston score that is obtained from non‐contrast ECG‐gated CT with precise settings in terms of kV and amperes [13]. After acquisition, usually the score is measured via manual or semi‐automated quantification. Then, following Coronary Artery Disease‐Reporting and Data System (CAD‐RADS) 2.0 indication, the patient can be categorized and personalized treatment can be applied (Figure 1) [14].

In the realm of CACS, AI contributes mainly to the post‐processing tasks and collaborates in cardiovascular disease prediction. Mostly, scientists focused on forging and validating a method to automatically detect and quantify CAC. The most advanced works rely on CNN AI implementation (Table 2).

**TABLE 2:** Summary of the most important papers involving AI and CACS by year.

Authors	Year of publication	AI implemented	Data evaluated	Main results
Gogin et al. [15]	2021	CNN with 3D U‐Net architecture	98 ECG‐gated CTs	CI: 0.951
Xu et al. [16]	2021	Deep learning (AI‐CACS software)	901 ECG‐gated CTs	Good correlation with manual CACS (*ρ* = 0.893 [*p* < 0.001]) with good category concordance (*ρ* = 0.893 [*p* < 0.001])
Ihdayhid et al. [17]	2023	3D CNN	1849 ECG‐gated CTs	Very strong correlation with manual score (Spearman's *r* = 0.90, 95% confidence interval [CI] 0.89–0.91, *p* < 0.001). 89% of the scans into the same risk category as human observers.
Yin et al. [18]	2024	Deep learning (AI‐CACS software)	Low‐dose non‐gated chest CT reconstructed at slice thickness of 1, 3, and 5 mm	Very high correlation with CACS obtained from ECG‐gated CTs (0.973, 0.941, and 0.834 [all *p* < 0.01]), respectively

Abbreviations: AI, Artificial intelligence; CACS, coronary artery calcium score; CI, Concordance Index; CNN, convolutional neural network; CT, computed tomography.

Gogin et al. used 783 unenhanced ECG‐gated CTs to train a five fully CNN with 3D U‐Net architecture to recognize and label CAC. After the training, 98 separate CTs were used to test the software. The software was shown to be fast and robust, with a Concordance Index of 98 [15].

Ihdayhid et al. built an AI‐based fully automated software to identify and quantify CAC on cardiac CT. A 3D CNN was developed to recognize high‐density voxels on 1849 multi‐vendor native ECG‐gated CTs. The model showed very high correlation and agreement when compared to manual segmentation. Moreover, the segmentation time was shorter compared to manual segmentation [17].

Even if CACS is widely used to stratify the patient's CAD risk, nowadays it necessarily passes via a distinct exam. To reduce radiation exposure new AI‐assisted technologies are being developed. Xu et al. used AI software (AI‐CACS) to automatically calculate the CACS in non‐gated chest CT. The software was trained on multi‐vendor, multi‐scanner non‐gated chest CT scans, thus not acquired for cardiac investigations. AI‐CACS was shown to have a good correlation with manual CACS obtained on ECG‐gaited CTs acquired on the same patients (*ρ* = 0.893 [*p* < 0.001]). Even if in some patients different values were found, there was good risk category concordance (kappa = 0.679 [*p* < 0.001]) [16]. Moreover, the diagnostic performance was excellent. These results could pave the way to non‐gated CT acquisition. Some patients (19.4%) were misclassified. This data is consistent with other studies and it is mostly due to motion artifacts or the inclusion of valvular calcification in the final CACS [19, 20].

Yin et al. moved forward by using AI‐CACS on non‐gated chest CT with different slice thicknesses (1, 3, and 5 mm). CACS automatic quantification exhibited excellent correlation (when compared with ECG‐gated CTA of the same patients 0.973, 0.941, and 0.834 [all *p* < 0.01], respectively), consistency and risk classification performance when evaluating 1‐ and 3‐mm‐slice‐thickness CT scans with the first type showing the best results. Again, the software was more prone to incorrect estimation in those patients having low‐risk CAD scores (1–100) [18].

CACS is nowadays widely used and usually represents the first step in CTA. The already in‐use software allows for a fast and reliable measurement. It is safe to assume that AI will provide help mostly in the post‐acquisition phase, especially in giving a feasible CACS in non‐gated, low‐dose scans. This implementation would simplify CHD screening by measuring CAC in all the non‐gated basal scans. Moreover, it could merge CAD screening into lung screening by taking one simple, non‐gated, lower‐dose CT acquisition.

## 3 CT Angiography

**FIGURE 2:**
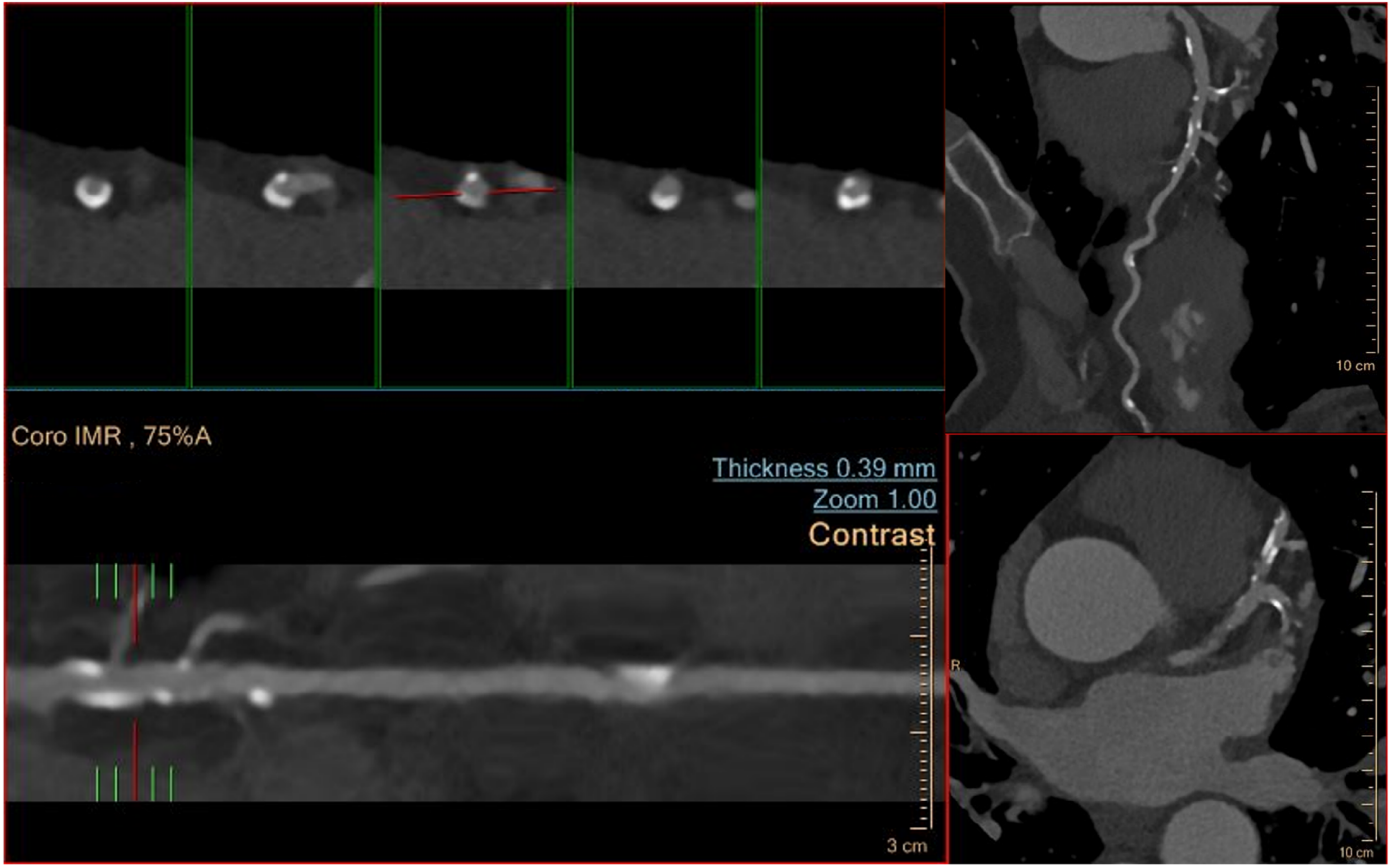
Example of CT angiography. In this example, multiple calcified plaques can be appreciated.

Nowadays, CTA has emerged as a cornerstone in CAD non‐invasive imaging. Bearing a very high specificity, CTA plays a great role as a gatekeeper for invasive coronary angiography (ICA). In addition, CTA has been proven to possess a prognostic value for MACE on top of the old cardiovascular risk factors [21]. In 2022 an expert consensus document (CAD‐RADS 2.0) was published by the Society of Cardiovascular Computed Tomography, the American College of Cardiology, the American College of Radiology, and the North American Society of Cardiovascular Imaging. In this document it is described in great detail the proper way to report the presence and the framework of the stenosis, the plaque burden, and the modifiers of coronary artery plaques (Figure 2). Moreover, it includes the importance of CT fractional flow reserve (FFR_CT_) and myocardial CT perfusion (CTP) [14]. Since its publication, the use of CAD‐RADS 2.0 has entered CTA reports and it is now widely used.

ML‐based algorithms have been developed to address every aspect of the CTA analysis after the acquisition. The application of ML includes image segmentation, plaque detection, texture analysis, and text analysis of radiology reports (Table 3).

**TABLE 3:** AI has been implemented in CTA evaluation in multiple ways. In this, tables are reported the most relevant ones by year.

Authors	Year of publication	AI implemented	Data evaluated	Main results
Kolossváry et al. [22]	2017	Radiomics‐based AI	30 CTAs with NRS plaques and 30 non‐NRS CTAs	20.6% of radiomic features were significantly different between NRS and non‐NRS plaques.
Zreik et al. [23]	2019	CNN	163 CTAs where the centerlines of the coronary arteries were extracted before the analysis	High accuracy to detect and characterize the plaque (0.77), and to highlight and determine the anatomical significance (0.80).
Kolossváry et al. [24]	2019	13 parameters, radiomics‐based ML	21 coronary arteries imagined ex vivo with CTA	The model outperformed visual assessment (AUC = 0.73, 95% CI 0.63–0.84 vs. AUC = 0.65, 95% CI 0.56–0.73, *p* = 0.04) in the identification of advanced atheromatous lesions.
Muscogiuri et al. [25]	2020	CNN	288 CTAs	To automatically classify a CTA into the correct CAD‐RADS category. Three models yielded sensitivity, specificity and accuracy between 47% and 82%, 58% and 91%, and 46% and 86%, respectively. Shorter time of analysis compared to human reading (530.5 ± 179.1 vs. 104.3 ± 1.4 s, *p*:0.01).
Lin et al. [26]	2022	ML	581 vessels	Accurate prediction of invasive FFR‐defined ischemia and impaired MBF by PET. AUC for the prediction of FFR‐defined ischemia significantly higher than visual assessment (0.92 vs. 0.84; *p* < 0.001) while comparable with FFR_CT_ (0.93; *p* = 0.34). AUC for the prediction of impaired MBF significantly higher than visual assessment (0.80 vs. 0.74; *p* = 0.02) while comparable with FFR_CT_ (0.77; *p* = 0.16).
Jonas et al. [27]	2022	CNN	303 CTAs	To verify if the model is consistent in CTAs acquired via multiple different parameters. No differences across all measured scanner, scan technique, patient preparation, contrast, and individual patient parameters on a per‐patient evaluation.
Yuan et al. [28]	2024	High‐level DL image reconstruction algorithm	210 patients were assigned to three groups with different contrast volume protocols: 0.7, 0.6, or 0.5 mL/kg	The reconstructions from all three groups resulted in good quality.

Abbreviations: AI, artificial intelligence; AUC, area under curve; CNN, convolutional neural network; CTA, computed tomography angiography; MBF, myocardial blood flow; ML, machine learning; NRS, napking‐ring sing; PET, positron emission tomography.

An early study by Zreik et al. investigated the performance of a multi‐task CNN algorithm on 163 CTAs. In this study, the centerline of the coronary arteries was extracted and used to obtain multiplanar reconstruction images. The algorithm was tested on two different tasks: to detect and characterize the plaque and to detect the stenosis and judge the anatomical significance. On both tasks, the algorithm had high accuracy (0.77 and 0.80, respectively) [23].

Muscogiuri et al. investigated a new deep CNN algorithm to automatically classify a CTA into the correct CAD‐RADS category. Three models (Model A, 1 and 2) were developed based on CAD‐RADS and put to the test. Sensitivity, specificity, and accuracy ranged between 47% and 82%, 58% and 91%, and 46% and 86%, respectively. Moreover, they demonstrated how the CNN‐based program showed a shorter average time of analysis compared to man‐made evaluation [25].

In a pivotal study, Kolossváry et al. compared a radiomics‐based ML assessment with a visual and histogram‐based evaluation of ex vivo coronary CTA cross‐sections to verify the presence of advanced atherosclerotic lesions. The 13 parameters ML model outperformed visual assessment (AUC = 0.73, 95% CI 0.63–0.84 vs. AUC = 0.65, 95% CI 0.56–0.73, *p* = 0.04) area of low attenuation and average HU in the identification of advanced atheromatous lesions [24]. The identification of high‐risk plaques plays a crucial role in the management of the patient. In a similar study, they discovered that 20.6% of the considered radiomic features were significantly different between coronary lesions with and without napkin ring signs. Moreover, the best radiomic predictor (short‐run low‐gray‐level emphasis) performed better than the best conventional parameter (mean plaque attenuation) (AUC 0.89 vs. 0.75) [22].

A total of 10 800 CTA images reviewed by an expert radiologist using CAD‐RADS were used by Paul et al. to train a DL model. The model detected ≥50% stenosis with an accuracy similar to the one achieved by senior radiologists [29].

Chen et al. developed a radiomic signature based on a data set of patients first undergoing CTA and then validated on intravascular ultrasound performed within 3 months. In the training, validation, and internal and external test sets the signature yielded an AUC of 0.81, 0.75, 0.80, and 0.77, respectively. A high radiomic signature (≥1.07) was independently linked with MACE [30].

In the assessment of myocardial fibrosis, CTA was demonstrated to be a valuable tool through late iodine enhancement (LIE) [31]. The added value of LIE comes with another acquisition, thus raising the ionizing burden. Penso et al. developed a DL algorithm that was able to detect myocardial fibrosis from early iodinated enhancement with an accuracy of 71% and an area under curve (AUC) of the receiver operating curve (ROC) of 76% [32].

One of the main problems when assessing the accuracy of an algorithm is external validation. A model based on single‐center or single‐vendor datasets could obtain high‐grade accuracy with similar parameters while being inefficient on different ones. Jonas et al. analyzed 303 CTAs with cloud‐based software that performs coronary segmentation, lumen and vessel wall determination, plaque quantification and characterization, and stenosis assessment. On per‐patient analysis there were no differences across all measured scanner, scan technique, patient preparation, contrast, and individual patient parameters [27].

Usually, CTA can be performed on the elderly population. In the aged population, the use of contrast enhancement may be conflictual due to kidney failure. With this type of patients, it would be appropriate to reduce contrast administration.

Yuan et al. evaluated the image quality of 210 patients who underwent CTA with either 0.7, 0.6, or 0.5 mL/kg. The images were post‐processed by a high‐level DL algorithm with a thickness of 0.625 mm. All the images were of good quality, despite the low contrast injection [28].

Lately, plaque assessment and evaluation have been explored for ischemia prediction. Lin et al. trained an ML algorithm to perform a score for the prediction of ischemia (FFR ≤ 80). Then, the ML score was used to predict impaired hyperemic myocardial blood flow (MBF) (≤2.30 mL/min per g) from positron emission tomography (PET) scans. The accuracy of the ML model was compared with CTA reads and noninvasive FFR_CT_. The ML algorithm accurately predicted invasive FFR‐defined ischemia and impaired MBF by PET. It performed superiorly to CTA stenosis evaluation and similar to FFR_CT_ (AUC of the ROC curves of 0.80, 0.74, and 0.77 respectively) [26].

Among all the tasks an imager can perform on cardiac CT, plaque analysis is the most time‐consuming one. Most of the available softwares allow for quick coronary reconstruction and stenosis assessment, but plaque analysis and more subtle information (e.g., peri‐coronary adipose tissue and presence of high‐risk modifiers) are still mostly performed by visual assessment. In the future, AI will focus on implementing high‐quality plaque classification and coronary reconstruction by lowering the radiation dose and the contrast agent administration.

## 4 Fractional Flow Reserve

**FIGURE 3:**
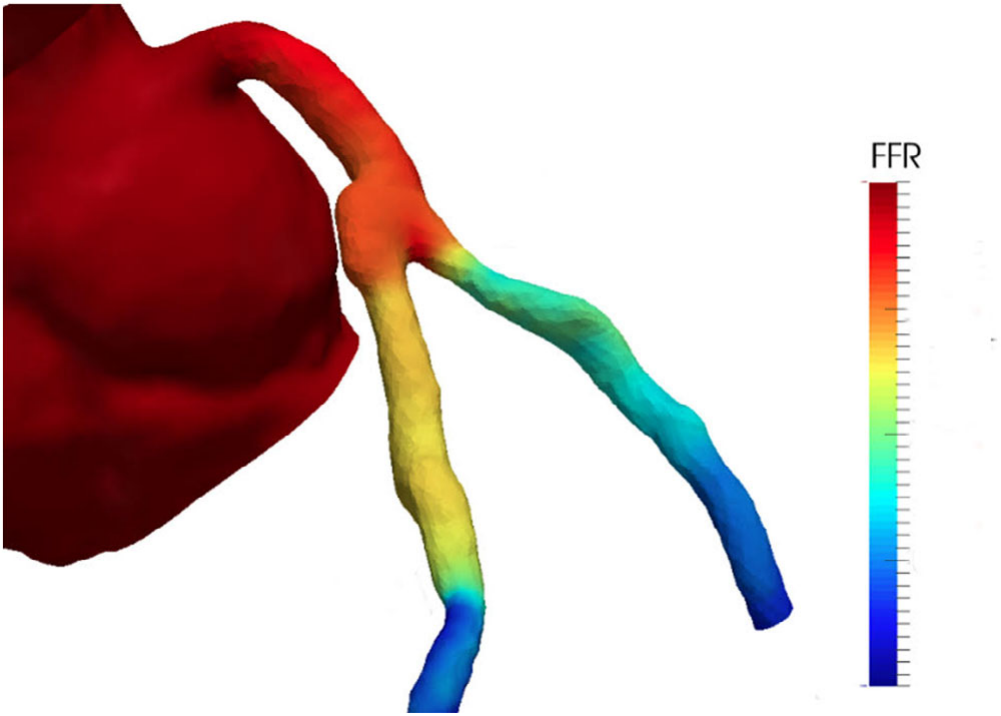
Rendering of a FFR_CT_ reconstruction. The results are color‐coded based on the grade of stenosis along the vessel.

One of the latest advances in CT technology is FFR_CT_. It is a novel, non‐invasive imaging tool for anatomical and physiological plaque assessment. It is based on computational fluid dynamics (CFD) modeling techniques and it renders an evaluation of blood‐flow impairment (Figure 3) [5]. Currently, only a limited number of vendors provide the option to assess FFR_CT_, and the post‐processing is typically performed externally by a third‐party service rather than on the workstation itself. Since its debut in 2016, the integration of AI in the measurement of FFR_CT_ has sprouted. Some studies have tried to fill this gap and provide innovative solutions (Table 4).

**TABLE 4:** AI implementation in the FFR_CT_ is quite recent. Thus, the literature is less expanded than for CACS and CTA.

Authors	Year of publication	AI implemented	Data evaluated	Main results
Takahashi et al. [33]	2023	DL	184 ECG‐gated CTA	For predicting FFR‐positive stenosis, the AUC of the model was far superior to the visual assessment one (0.756 vs. 0.574 (*p* = 0.055)).
Giannopoulus et al. [34]	2023	On‐site DL algorithm	59 CTAs	Strong correlation with invasive FFR (*r* = 0.81). At a cutoff of 0.80 or less, FFR‐CT had 95.9% accuracy, 93.5% sensitivity, and 97.7% specificity. Mean analysis time = 8 min per CTA.
Peters et al. [35]	2024	AI (FFR_AI_)	37 patients with 39 intermediate‐grade stenosis on CTA	Compared to invasive FFR, FFR_AI_, and FFR_CT_ showed moderate to high sensitivity (91% vs. 82%), specificity (82% vs. 75%), positive predictive value (67% vs. 56%), and negative predictive value (96% vs. 91%). Diagnostic accuracy did not differ significantly between FFR_AI_ (85%) and FFR_CT_ (77%) (*p* = 0.12)
Guo et al. [36]	2024	ML	110 CTAs	Compared to invasive FFR, ML‐based FFR_CT_ algorithm and myocardial perfusion reserve (MPR) had similar AUC, sensitivity, specificity, and accuracy: 0.90 vs. 0.89, 89% vs. 79%, 87% vs. 84%, and 88% vs. 83%, respectively

Abbreviations: AI, Artificial intelligence; AUC, area under curve; CNN, convolutional neural network; CTA, computed tomography angiography; DL, deep learning; FFR, fractional flow reserve; ML, machine learning.

Takahashi et al. developed a DL model for predicting FFR‐positive stenosis in coronary arteries, especially for the calcified plaques. The results of the DL model were compared to classical visual assessment. The AUC of the ROC curve was 0.756, far superior to the one obtained from visual assessment, based on the presence of a >70% stenosis (0.574) [33].

A study by Sun et al. built a BPNN to predict stenosis resistance by entering vascular geometric parameters and blood flow. They then compared the BNPP model with existing 3D CFD software. The BPNN method had an accuracy not significantly different from the 3D CFD software, but it was faster and simpler to use [11].

In a proof of concept study, Peters et al. trained an FFR_AI_ model with CTA images of 500 stenotic vessels in 413 patients, using FFR measurements as the ground truth. Then they retrospectively included 37 patients with 39 intermediate‐grade stenosis on CTA that underwent also invasive FFR and FFR_CT_ measurements. FFR_AI_ and FFR_CT_ showed respectively moderate to high sensitivity (91% vs. 82%), specificity (82% vs. 75%), positive predictive value (PPV) (67% vs. 56%), and negative predictive value (NPV) (96% vs. 91%). Diagnostic accuracy did not differ significantly between FFR_AI_ (85%) and FFR_CT_ (77%) (*p* = 0.12) [35].

To increase the implementation of an algorithm in the workstation, Giannopoulus et al. developed and implemented a high‐speed on‐site deep learning–based FFR_CT_ algorithm. In this retrospective study, 59 patients who underwent both CTA and invasive FFR assessment were included. The AI algorithm included a 3D computational flow dynamics model. Hemodynamic evaluations from the algorithm were compared to invasive FFR and showed a strong correlation (*r* = 0.81). FFR_CT_ had an AUC of 0.975. Considering 0.80 as a cut‐off, the model had 94.7% sensitivity, 95.0% specificity, and 94.9% accuracy. The mean analysis time was around 8 min and the interobserver and intraobserver agreement were good to excellent [34].

Guo et al. assessed the performance of an ML‐based FFR_CT_ algorithm and myocardial perfusion reserve (MPR) assessed via cardiac magnetic resonance perfusion when compared to invasive FFR. The study involved 110 patients, and 36 significant stenosis were detected. MPR and the ML algorithm had similar AUC, sensitivity, specificity, and accuracy: 0.90 versus 0.89, 89% versus 79%, 87% versus 84%, and 88% versus 83%, respectively [36].

Among the different tools CT offers for cardiac images, FFR_CT_ is one of the less accessible. The very few available software led to an increased demand/offer ratio thus raising the prices for every single evaluation. Moreover, the concept of non‐invasive individuation of hemodynamically significant stenosis is gaining importance in the reporting of CTA images [14]. The validation of new and fast AI algorithms would provide the most benefit to the imagers and will help deliver personalized treatments.

## 5 Computed Tomography Perfusion

**FIGURE 4:**
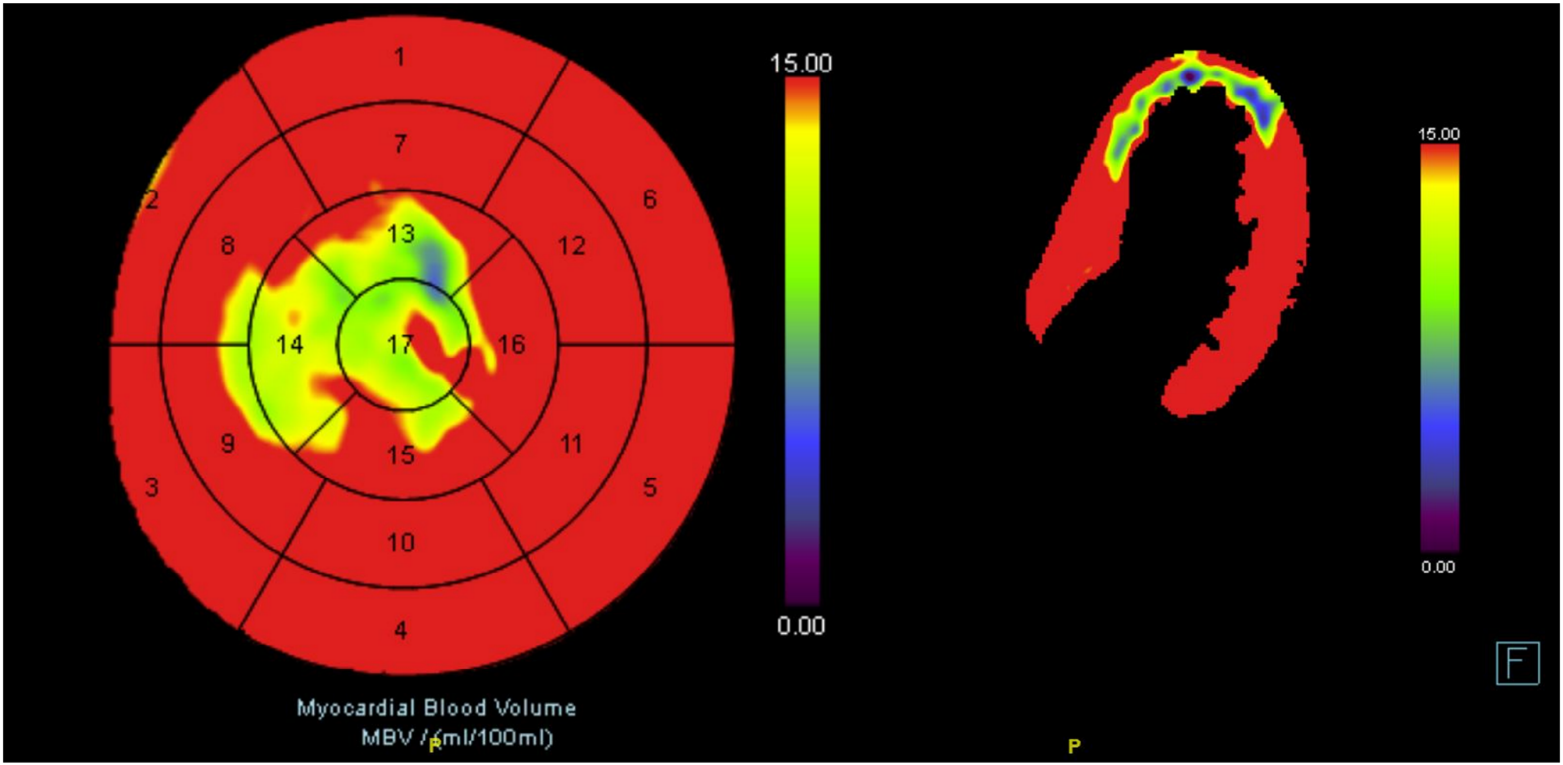
Example of myocardial dynamic CTP analysis showing a reduction in blood volume in different myocardial segments.

Myocardial CTP represents a stress‐induced CT acquisition that adds diagnostic and prognostic value to CTA [37, 38]. Particularly, CTP adds functional information to standard CTA thus providing better guidance to invasive intervention. Static CTP provides a single snapshot of the iodinated contrast agent distribution in the myocardium. Perfusion defects are assessed in a qualitative way by highlighting hypo‐enhanced areas. Dynamic CTP allows non‐invasive quantification of myocardial blood flow (MBF) from the time‐attenuation curves of both the blood pool and the myocardium (Figure 4) [39].

In the last decade, CTP technical aspects have been refined and nowadays CTP is a widely proposed exam. Since CTP represents one of the newest explored tools, only a few studies have tried to implement AI in the post‐processing of CTPs to shorten the time of reporting and to enhance perfusion defect recognition (Table 5).

**TABLE 5:** As per FFR_CT_, although promising a vast literature regarding AI and CTP is lacking. In this table, the most important papers are reported by year.

Authors	Year of publication	AI implemented	Data evaluated	Main results
Han et al. [40]	2018	ML	252 CTAs with suspected CAD	Boost of the discrimination and reclassification of possibly ischemic patients (net reclassification improvement: 0.52, *p* value < 0.001)
Takafuji et al. [41]	2022	DL	30 low‐tube voltage CTAs	The algorithm rendered good‐quality images
Muscogiuri et al. [42]	2022	DL	112 CTAs	Compared to invasive assessment for hemodynamically significant CAD, the AUC of CTA + CTP_stress_, CTA + DL_rest_, and CTA + DL_stress_ were 87%, 96%, and 98%, respectively

Abbreviations: AI, Artificial intelligence; AUC, area under curve; CAD, coronary artery disease; CTA, computed tomography angiography; CTP, computed tomography perfusion; DL, deep learning; ML, machine learning.

In a study conducted by Muscogiuri et al., a DL algorithm was tested to predict hemodynamically significant CAD by using a rest dataset of myocardial CTP compared to invasive FFR. The accuracy of the algorithm was tested on 112 consecutive symptomatic patients who underwent CTA, rest, and stress CTP and invasive FFR for stenosis ranging between 30% and 80%. The sensitivity, specificity, NPP, PPV, accuracy, and AUC of rest and stress DL‐CTP were similar thus proving that a DL approach on rest CTP is feasible and accurate and can be used as a gatekeeper to stress CTP. Moreover, the DL algorithm proved to be faster than human analysis when evaluating CTP (39.2 ± 3.2 vs. 379.6 ± 68.0 s, *p* < 0.001) [42].

Takafuji et al. implemented a DL reconstruction model to decrease image noise and image quality of dynamic CTPs with low tube voltage in comparison with hybrid iterative reconstruction while maintaining quantification of MBF. The main goal of this study was to assess the feasibility of the model while lowering the dose exposure. The model proved to be able to provide good‐quality images on 30 consecutive patients [41].

Kim et al. assessed the accuracy of novel U‐net‐based deep CNN models to segment the aorta and the myocardium after dynamic CTP acquisition and to identify perfusion defects. Mean dice scores were 0.94 (±0.07) and 0.86 (±0.06) for the aorta and the myocardium, respectively. The identification of perfusion defects had an AUC of the ROC curves of 0.959 for LAD, 0.949 for RCA, and 0.957 for LCX [43].

Han et al. tested a ML‐trained algorithm to evaluate the rest of CTP and provide a prediction on significant ischemia. They then compared the results over normal CTA stenosis assessment. The ML‐based algorithm was tested on 252 patients with suspected CAD. The use of the algorithm boosted the discrimination and reclassification (net reclassification improvement: 0.52, *p* value < 0.001). In particular, implementing resting CTP to CTA incremented the diagnostic AUC for significant stenosis from 0.68 (95% CI, 0.620.74) to 0.75 (95% CI, 0.69–0.81) [40].

The potential to integrate functional assessment into what has traditionally been considered a predominantly qualitative examination presents a compelling opportunity to enhance both diagnostic and prognostic accuracy [44]. This is particularly relevant for myocardial CTP, which offers the ability to evaluate not only anatomical but also functional parameters. However, it is important to acknowledge the two primary limitations associated with CTP: the elevated exposure to ionizing radiation and the increased complexity and duration of image analysis and reporting.

Recent studies have explored methods for reducing radiation dose, including the application of ML algorithms in the post‐processing workflow. These algorithms have demonstrated potential in optimizing image reconstruction and reducing noise, thereby allowing for dose reduction without compromising diagnostic accuracy. Additionally, emerging models are investigating the feasibility of conducting a “rest‐only” CTP protocol, which would eliminate the need for a stress‐phase acquisition, thereby reducing the overall radiation dose and simplifying the examination protocol.

Moreover, the incorporation of AI‐driven algorithms holds promise in assisting radiologists with image analysis, potentially decreasing reporting time by automating certain aspects of image interpretation. These advancements could contribute to faster, more accurate reporting while maintaining high diagnostic standards. The convergence of these technologies positions CTP as a highly promising tool in cardiac imaging provided that these challenges are effectively addressed [41, 42].

## 6 Epicardial Adipose Tissue

Epicardial adipose tissue is the portion of fat tissue between the myocardium and pericardium visceral layer. It has been proven the presence of stromal, immune cells, inflammatory cells, and nervous tissue within the tissue [45] and it has been proposed how EAT can play a role in the protection of toxic levels of free fatty acids [46]. Moreover, Monti et al. depicted how EAT significantly changes after the administration of one of the most cardiotoxic chemotherapy [47].

In recent years, a limited number of studies on AI applications for evaluating EAT have been published. EAT is commonly assessed using non‐contrast scans, but its segmentation can be challenging and time‐consuming, particularly with non‐gated CT images (Table 6). Foldyna et al. tried to assess whether a DL algorithm could automatically segment EAT volume and its prognostic value for all‐cause and cardiovascular mortality. The model was tested on 24 090 CTs from heavy‐smoker patients and was shown to be reliable. EAT volume was demonstrated to be independently associated with all‐cause mortality [48]. A similar conclusion was obtained by West et al. when a DL model was used on 3720 CTAs to assess EAT volume. The concordance correlation coefficient between machine and human assessment was 0.970 [49].

**TABLE 6:** Summary of the most important papers involving AI and EAT by year.

Authors	Year of publication	AI implemented	Data evaluated	Main results
Eisenberg et al. [50]	2020	DL	2068 unenhanced CTs	The fully automated EAT segmentation provided prognostic value for MACE
Zhang et al. [51]	2022	ML	200 unenhanced and 300 enhanced CTs	High AUC for predicting atrial fibrillation (0.92 and 0.85, respectively)
West et al. [49]	2023	DL	1811 enhanced CTs	Concordance correlation coefficient between AI and human of 0.97
Foldyna et al. [48]	2024	DL	24 090 unenhanced CTs of heavy‐smoker patients	The model was accurate in automatically segments EAT volume
Hu et al. [52]	2024	DL	400 low‐dose CAC exams (unenhanced)	The high‐risk group had a hazard ratio for MACE 2.4 higher than that of the low‐risk group

Abbreviations: AI, artificial intelligence; AUC, area under curve; CT, computed tomography; DL, deep learning; EAT, epicardial adipose tissue; MACE, major adverse cardiovascular events; ML, machine learning.

Eisenberg et al. created a DL software to automatically segment EAT and measure its volume and mean attenuation. The study included 2068 asymptomatic subjects with a follow‐up period of 14 ± 3 years. It showed that EAT volume and attenuation quantification by DL can provide prognostic value for MACE in asymptomatic patients [50].

Hu et al. studied low‐dose CACS exams from 400 patients. One hundred forty‐eight features were extracted and analyzed via a DL algorithm. Among them, 15 features showed significant improvement in MACE prediction, as the identified high‐risk group showed a hazard risk 2.4 times that of the low‐risk group [52].

Some evidence undermined the biological link between EAT and the occurrence and progression of atrial fibrillation (AF). Zhang et al. tested an ML approach to detect the presence of AF through EAT feature analysis in contrast‐enhanced (200 patients) and non‐enhanced (300 patients) CTs. The AUC for the first and second groups were 0.92 (95% CI: 0.84–1.00) and 0.85 (0.77–0.92), respectively [51].

Within the pericardium, apart from the myocardium and the coronary arteries lies the EAT. Its role is recently being rediscussed as new evidences are emerging [8]. AI is addressing key challenges, including the time‐intensive process of EAT segmentation, while also introducing tools for improved risk stratification, such as radiomic feature analysis. Future studies should prioritize robust, deep learning‐based algorithms capable of functioning across multiple imaging platforms. Additionally, the relevance of these approaches in predicting MACE warrants careful evaluation.

## 7 Conclusions

Radiology has transformed dramatically in recent decades due to rapid technological advancements, leading to the widespread adoption of non‐invasive imaging techniques and reshaping diagnostic workflows. Cardiac imaging, in particular, has grown significantly, enhancing both diagnostic and prognostic assessment of CAD and increasing demand for advanced imaging. Recently, AI has made substantial progress in cardiac CT, especially in the post‐processing phase, where sophisticated algorithms improve image quality, reduce radiation exposure, and automate image interpretation. Integrating AI into cardiac CT workflows optimizes efficiency and accuracy, offering a new standard in assessing complex cardiovascular conditions.

AI's primary applications in anatomical evaluation in cardiac imaging include CACS, CTA, and EAT. A promising development for CAC involves integrating CACS into non‐gated CT scans, which could enable routine screening during standard CT exams. Plaque assessment, however, remains challenging due to dataset heterogeneity, which affects algorithm performance and limits external validation. The implementation of DL algorithms into EAT automatic segmentation and measurement have demonstrated good accuracy thus paving the way for its integration in risk assessment. Functional assessments like FFR_CT_ and CTP are less common but offer the potential for ischemia evaluation; AI could reduce acquisitions and radiation exposure in these costly exams. Ultimately, AI could deliver comprehensive models that integrate CAC, plaque assessment, EAT, FFR_CT_, and CTP, supporting improved prognostic value and more personalized treatment strategies [44, 53].

## Conflicts of Interest

The authors declare no conflicts of interest.

## Data Availability

Data sharing not applicable to this article as no datasets were generated or analyzed during the current study.
